# Inhibition of melanoma growth in a subcutaneous model using ultrasound with low duty cycle

**DOI:** 10.1186/2050-5736-3-S1-P55

**Published:** 2015-06-30

**Authors:** Yuta Ando, Kelsie Timbie, Ji Song, James Larner, Richard Price, Kumari Andarawewa

**Affiliations:** 1 University of Virginia, Charlottesville, Virginia, United States

## Background/introduction

Melanoma has a 5-year survival rate of <10%, primarily due to therapeutic resistance and immune tolerance. Durable responses to therapy of metastatic melanoma are enhanced when the anti tumor immune response is activated. FUS is an emerging non-invasive treatment modality for localized treatment of cancers. Traditionally FUS has been used exclusively for thermal ablation of the target sites; biological responses associated with both thermal and mechanical damage have not been investigated. Damaged tumor cells have release endogenous danger signals, which stimulate an immune response, suggesting that the patient’s dying cancer cells may serve as a therapeutic vaccine which stimulates an antitumor immune response. Specifically, we hypothesize that FUS will augment tumor antigen release and danger signals to mediate rejection of both FUS treated and untreated tumors.

## Methods

Studies using C57BL6 mice inoculated subcutaneously with B16 melanoma cells were approved by the institutional animal care and use committee. When tumors became palpable, 100 cycles of 1MHz ultrasound was applied to the tumor every 5 seconds for 5, 10 or 20 minutes. Following treatment, tumor growth, survival, necrosis, and apoptosis were measured. Tumor volume was evaluated by taking daily measurements with digital calibers. Standard H & E staining was performed to evaluate histological changes that may have occurred as a result of the ultrasound exposures. The detection of apoptotic cells was performed using staining for Caspase 3.

## Results and conclusions

Tumors harvested 3 days after treatment were analyzed using H & E and caspase 3 staining. Staining with H & E indicated that significantly more necrotic tissue was present in all tumors treated with ultrasound. Staining for Caspase 3 exhibited more extensive apoptosis in all treatment groups when compared to untreated tumors.

Furthermore we found that ultrasound treatment dramatically decreased tumor size.

1 MHz ultrasound with low duty cycle can inhibits melanoma tumor. We will use this platform to characterize the immune response in treated tumors. We also plan to combine microbubbles with ultrasound to see if there is a synergistic effect on inhibition of tumor growth.

